# Caspase-9 mediates Puma activation in UCN-01-induced apoptosis

**DOI:** 10.1038/cddis.2014.461

**Published:** 2014-10-30

**Authors:** C Nie, Y Luo, X Zhao, N Luo, A Tong, X Liu, Z Yuan, C Wang, Y Wei

**Affiliations:** 1 The State Key Laboratory of Biotherapy and Cancer Center/Collaborative Innovation Center of Biotherapy, West China Hospital and College of Life Science, Sichuan University, No. 17 People's South Road, Chengdu 610041, People's Republic of China; 2 Nankai University School of Medicine/Collaborative Innovation Center of Biotherapy, Tianjin 300071, People's Republic of China

## Abstract

The protein kinase inhibitor 7-hydroxystaurosporine (UCN-01) is one of the most potent and frequently used proapoptotic stimuli. The BH3-only molecule of Bcl-2 family proteins has been reported to contribute to UCN-01-induced apoptosis. Here we have found that UCN-01 triggers Puma-induced mitochondrial apoptosis pathway. Our data confirmed that Akt-FoxO3a pathway mediated Puma activation. Importantly, we elucidate the detailed mechanisms of Puma-induced apoptosis. Our data have also demonstrated that caspase-9 is a decisive molecule of Puma induction after UCN-01 treatment. Caspase-9 mediates apoptosis through two kinds of feedback loops. On the one hand, caspase-9 enhances Puma activation by cleaving Bcl-2 and Bcl-xL independent of caspase-3. On the other hand, caspase-9 directly activated caspase-3 in the presence of caspase-3. Caspase-3 could cleave XIAP in an another positive feedback loop to further sensitize cancer cells to UCN-01-induced apoptosis. Therefore, caspase-9 mediates Puma activation to determine the threshold for overcoming chemoresistance in cancer cells.

The apoptosis pathway is closely related to the Bcl-2 family proteins in which antiapoptotic members sequester multidomain proapoptotic proteins, thereby inhibiting their active role in apoptosis. In contrast, BH3-only proteins that are considered stress sensors can dissociate Bax-like proteins from their antiapoptotic sequestrators, and thus leading to apoptosis.^1^

The expression of Bcl-2 family proteins is regulated during carcinogenesis,^1^ and the expression of both the Bcl-2 and Bcl-xL antiapoptotic proteins is associated with resistance to antitumor agents such as cisplatin (CP).^2^ The inhibition of the protective function of antiapoptotic Bcl-2 members can either restore the normal apoptotic process in cancer cells or circumvent resistance to chemotherapy.^3,4^ In this regard, enhanced expression of BH3-only proteins can effectively bind the antiapoptotic members and prevent the function of these proteins.

Some reports suggest that the BH3-only protein Puma has important roles in p53-dependent and -independent apoptosis in human cancer cells and mediates cell death through the Bcl-2 family proteins Bax/Bak and the mitochondrial pathway.^5,6^ Our studies also reveal that Puma upregulation induces cell apoptosis in chemoresistant ovarian cancer cells,^7,8^ confirming the requisite role of Puma in chemosensitivity.

7-Hydroxystaurosporine (UCN-01) is a protein kinase C-selective inhibitor that is successfully used in phase I and II clinical trials.^9,10^ As a modulator, UCN-01 enhances the cytotoxicity of other anticancer drugs such as DNA-damaging agents and antimetabolite drugs by putative abrogation of G2- and/or S-phase accumulation induced by these anticancer agents.^11^ As a single agent, UCN-01 exhibits two key biochemical effects, namely accumulation of cells in the G1 phase of the cell cycle and induction of apoptosis.^12^ Both these effects may be important for its anticancer activity. Previous studies have demonstrated that UCN-01 potently decreased the levels of activated the phosphorylation level of Akt (p-Akt) in *in vitro* or in *in vivo* systems.^12, 13, 14^ Some researchers have also approved that UCN-01 can modulate Bcl-2 family members to potentiate apoptosis in cancer cells.^15,16^ These reports suggest that Akt and Bcl-2 family proteins may be the potent targets of UCN-01 to trigger cancer cell apoptosis.

In this study, we also investigate the role of Puma in UCN-01-induced apoptosis and confirm that p53-independent Puma induction is pivotal for the anticancer effects of UCN-01. Moreover, we first elucidate the detailed mechanism of Puma-induced apoptosis after UCN-01 treatment. We found that Puma expression mediated caspase-9 and caspase-3 activation. Among the caspase proteins, caspase-9 has a key role in Puma-induced apoptosis. Our data demonstrated that caspase-9 could mediate Puma-induced apoptosis through two feedback pathways. On the one hand, activated caspase-9 was initiated followed by caspase-3 activity, and activated caspase-3 cleaved XIAP in a positive feedback loop to strengthen Puma expression. On the other hand, caspase-9 itself cleaved antiapoptotic Bcl-2 and Bcl-xL to positively enhance Puma induction. These results provide the detailed mechanistic insight into therapeutic response to UCN-01 and the theoretical basis for its applications.

## Results

### Puma is induced by UCN-01 in an Akt-FoxO3a-dependent and p53-inpendent manner

**Figure 1:**
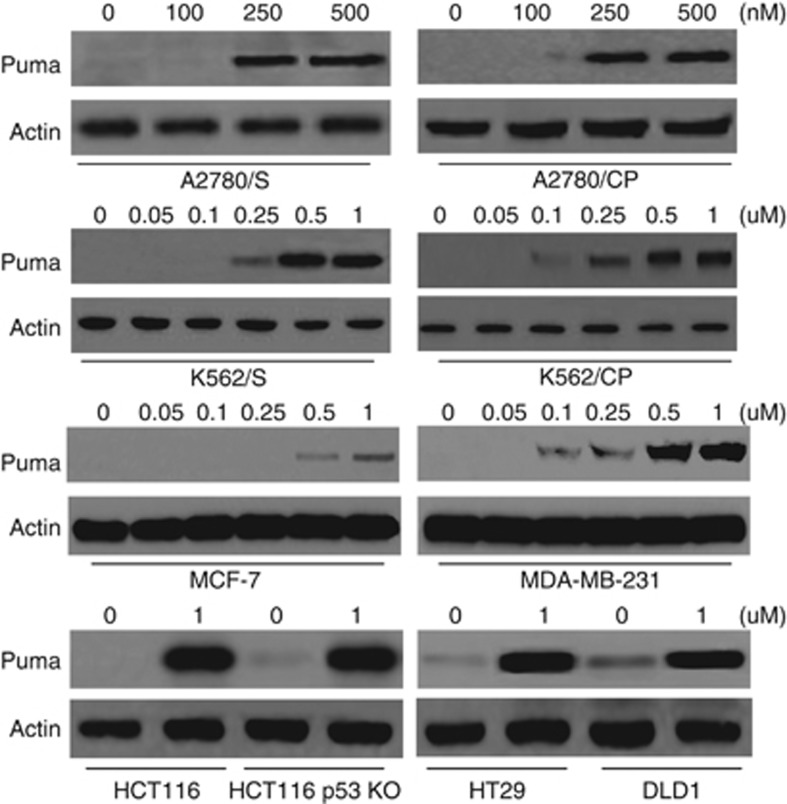
Puma induction by UCN-01 in cancer cells. Cells were treated with the indicated concentrations of UCN-01 for 24 h, and then collected and lysed for detection of Puma expression. *β*-Actin was used as a protein loading Ctrl. Representative results of three experiments with consistent results are shown

Our study revealed that UCN-01 treatment resulted in Puma induction in a variety of tumor cells, such as human colon cells (HCT116, HCT116 p53 KO, HT29 and DLD1), human ovarian cells (A2780/S and A2780/CP), leukemia cells (K562/S and K562/CP) and breast cancer cells (MCF-7 and MDA-MB-231) (Figure 1). These results further demonstrated that Puma induction was not dependent on p53 activation and revealed that UCN-01 could trigger Puma expression in different cancer cell lines regardless of their chemosensitivity.

**Figure 2:**
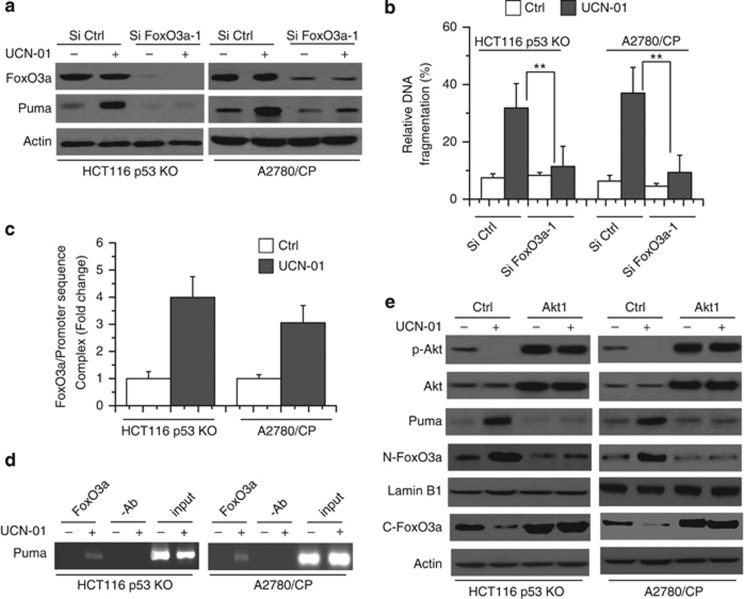
Akt-FoxO3a pathway directly mediates Puma induction following UCN-01 treatment. (**a**) Cells were transfected with Si Ctrl or Si FoxO3a-1 for 48 h, and then treated with UCN-01 (1 *μ*M for HCT116 p53 KO and 250 nM for A2780/CP) for 24 h. Treated cells were lysed to detect protein level by western blot. (**b**) Cells were transfected with Si Ctrl or Si FoxO3a-1 for 48 h, and then treated with UCN-01 (2 *μ*M for HCT116 p53 KO and 250 nM for A2780/CP) for 48 h. Cell apoptosis was quantitatively detected by a Cell Death ELISA Kit as described in the Materials and Methods section. Graphs show the results of quantitative analyses (*n*=3, mean±S.D., **P<0.01). (**c**) Quantification of FoxO3a association with the Puma promoter. ChIP was performed with anti-FoxO3a polyclonal antibody incubated with protein G-coated magnetic beads at 4 °C overnight with rotation. DNA fragments co-immunoprecipitated with the target protein FoxO3a were subjected to QRT-PCR. QRT-PCR assays were conducted on cells that were either left untreated (con) or treated with UCN-01 (1 *μ*M for HCT116 p53 KO and 250 nM for A2780/CP) for 24 h. Numbers on the y axis represent the levels of FoxO3a association with the Puma promoter region after normalizing to Ct values from input samples. Data shown are means±S.D. from three independent experiments. (**d**) ChIP was carried out on fixed cancer cells following 12 h of UCN-01 (1 *μ*M for HCT116 p53 KO and 250 nM for A2780/CP) treatment. An antibody specific for FoxO3a or no antibody was used to show specificity. PCR was carried out using primers surrounding the FoxO3a binding sites in the Puma promoter. (**e**) Cancer cells were transfected with Ctrl and constitutively active Akt1 (T308D and S473D) vector for 48 h and then treated with UCN-01 (1 *μ*M for HCT116 p53 KO and 250 nM for A2780/CP) for 24 h. Treated cells were lysed for detection. For FoxO3a nuclear translocation analysis, cells were incubated with UCN-01 and subjected to subcellular fractionation as described in the Materials and Methods section. Nuclear FoxO3a is referred to N-FoxO3a and cytosolic FoxO3a is referred to C-FoxO3a. Lamin B1 was used as a nuclear marker. Data are representative of at least three independent experiments

We further studied whether Akt-FoxO3a pathway was involved in regulating Puma induction in our experimental systems. As illustrated in Figure 2a and Supplementary Figure 1A, FoxO3a small interfering RNA (siRNA) efficiently decreased FoxO3a expression and Puma induction, as well as cell apoptosis after UCN-01 treatment. We then determined whether FoxO3a regulates Puma expression. We used the chromatin immunoprecipitation (ChIP) assay to detect the interactions between FoxO3a and the Puma promoter, as described previously.^7^ Our results revealed that FoxO3a could act on the Puma promoter after UCN-01 treatment (Figure 2b). Moreover, gel analysis also proved that the binding of FoxO3a to the Puma promoter was significantly enhanced after treatment. These results suggest that FoxO3a can directly bind to the Puma promoter to activate its transcription following UCN-01 treatment.

We then determined whether Akt mediates FoxO3a-induced Puma expression. Transfection with Akt1 vector increased p-Akt and total Akt expression. Meanwhile, Akt1 transfection decreased FoxO3a nuclear translocation and increased its cytosolic location after UCN-01 treatment. Furthermore, Akt1 overexpression suppressed Puma expression (Figure 2c). Therefore, the induction of Puma by FoxO3a following UCN-01 treatment was to be mediated through Akt inhibition.

### Puma mediates UCN-01-induced apoptosis

**Figure 3:**
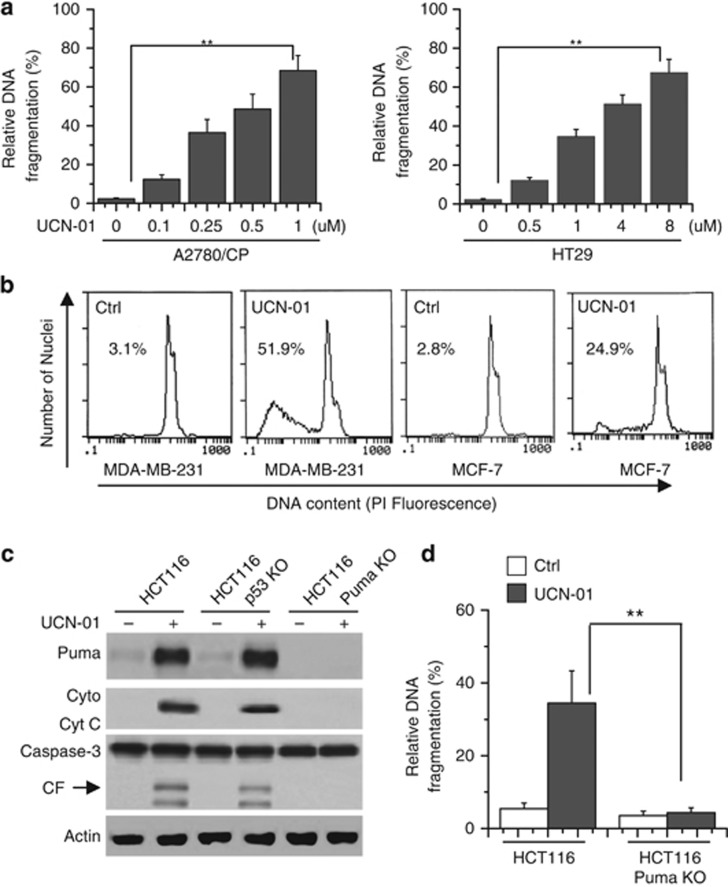
Puma is required for the chemosensitization effects of UCN-01 and mediates apoptosis. (**a**) Cells were treated with indicated concentrations of UCN-01 for 48 h. Cells were collected and lysed to detect apoptosis. Cell apoptosis was quantitatively detected by a Cell Death ELISA Kit as described in the Materials and Methods section. Graphs show results of quantitative analyses (*n*=3, mean±S.D., **P<0.01). (**b**) Cells were treated with 2 *μ*M UCN-01 for 48 h, and then collected for PI staining. Sub-G1 cells and apoptotic cells, respectively, were assessed by flow cytometry. Representative results of three experiments with consistent results are shown. (**c**) Cells were treated with 2 *μ*M UCN-01 for 48h. Cells were collected and lysed for western blot analysis. CF means the cleaved fragment of caspase-3. *β*-Actin was used as a protein loading Ctrl. (**d**) HCT116 or HCT116 Puma KO cells were treated with 2 *μ*M UCN-01 48 h. Cells were lysed to detect apoptosis by a Cell Death ELISA Kit as described in the Materials and Methods section. Graphs show results of quantitative analyses (*n*=3, mean±S.D., **P<0.01)

We first determined the apoptotic effect of UCN-01 in various cancer cell lines. Cells were treated with UCN-01 and apoptosis was confirmed by a DNA fragmentation ELISA assay at indicated concentrations. These results revealed that UCN-01 effectively induced apoptosis in A2780/CP and HT29 cells (Figure 3a). Flow cytometry analysis with PI staining further demonstrated the effect of UCN-01 on apoptosis in breast cancer cells (Figure 3b). The other cancer cells showed the same results (data no shown).

We then investigate the role of Puma in UCN-01-induced apoptosis. Puma knockout (KO) or knockdown (KD) by siRNA abrogated UCN-01-induced cytochrome c (Cyt *c*) release and caspase-3 and -9 activation (Figure 3c and Supplementary Figure 1B). DNA fragmentation ELISA assay also affirmed that Puma mediated UCN-01-induced apoptosis (Figure 3d and Supplementary Figure 1C).

**Figure 4:**
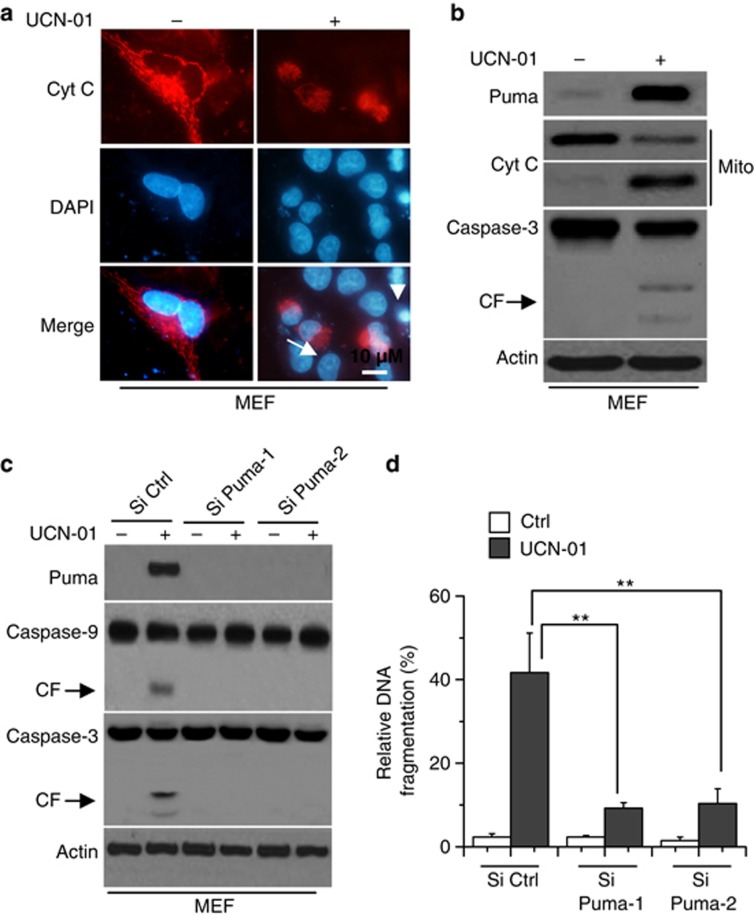
Puma is necessary for UCN-01-induced MEF cell apoptosis. (**a**) MEF cells were treated with 6 *μ*M UCN-01 for 48 h, and then cells were fixed and immunostained for Cyt *c* as described in the Materials and Methods section. Nuclei were counterstained with DAPI (4',6-diamidino-2-phenylindole). Arrow indicates the release of Cyt *c*. Arrowhead refers to nuclear condensation and fraction. (**b**) MEF cells were treated with 6 *μ*M UCN-01 for 48 h, and then lysed for western blot analysis. *β*-Actin was used as a protein loading Ctrl. (**c**) MEF cells were transfected with Si Ctrl or Si Puma-1, 2 for 48 h, and then treated with 6 *μ*M UCN-01 for 48 h. Treated cells were collected for western blot analysis. Representative results of three experiments with consistent results are shown. (**d**) As described in (**c**), treated cells were collected for detection of cell apoptosis. Graphs show results of quantitative analyses (*n*=3, mean±S.D., **P<0.01)

Further experiments demonstrated that UCN-01 induced the release of Cyt *c* and nuclear condensation and fractions in murine embryonic fibroblast (MEF) cells (Figures 4a and b). Moreover, UCN-01 also triggered Puma expression and caspase-3 cleavage (Figure 4b). Puma KD by siRNA confirmed that Puma is necessary for caspase-3 and -9 activation and subsequent cell apoptosis induced by UCN-01 (Figures 4c and d).

### Caspase-9 regulates Puma and caspase-3 activation in apoptosis

Next, we detect the downstream events of Puma activation. We found that the apoptotic rate of MCF-7 was apparently lower than that of MDA-MB-231 after UCN-01 treatment (Figure 3b). Because MCF-7 lacks caspase-3 expression,^17^ we speculate that the other molecules are involved in Puma-induced apoptosis in addition to caspase-3. It is likely that there is a caspse-3-independent pathway in apoptosis.

**Figure 5:**
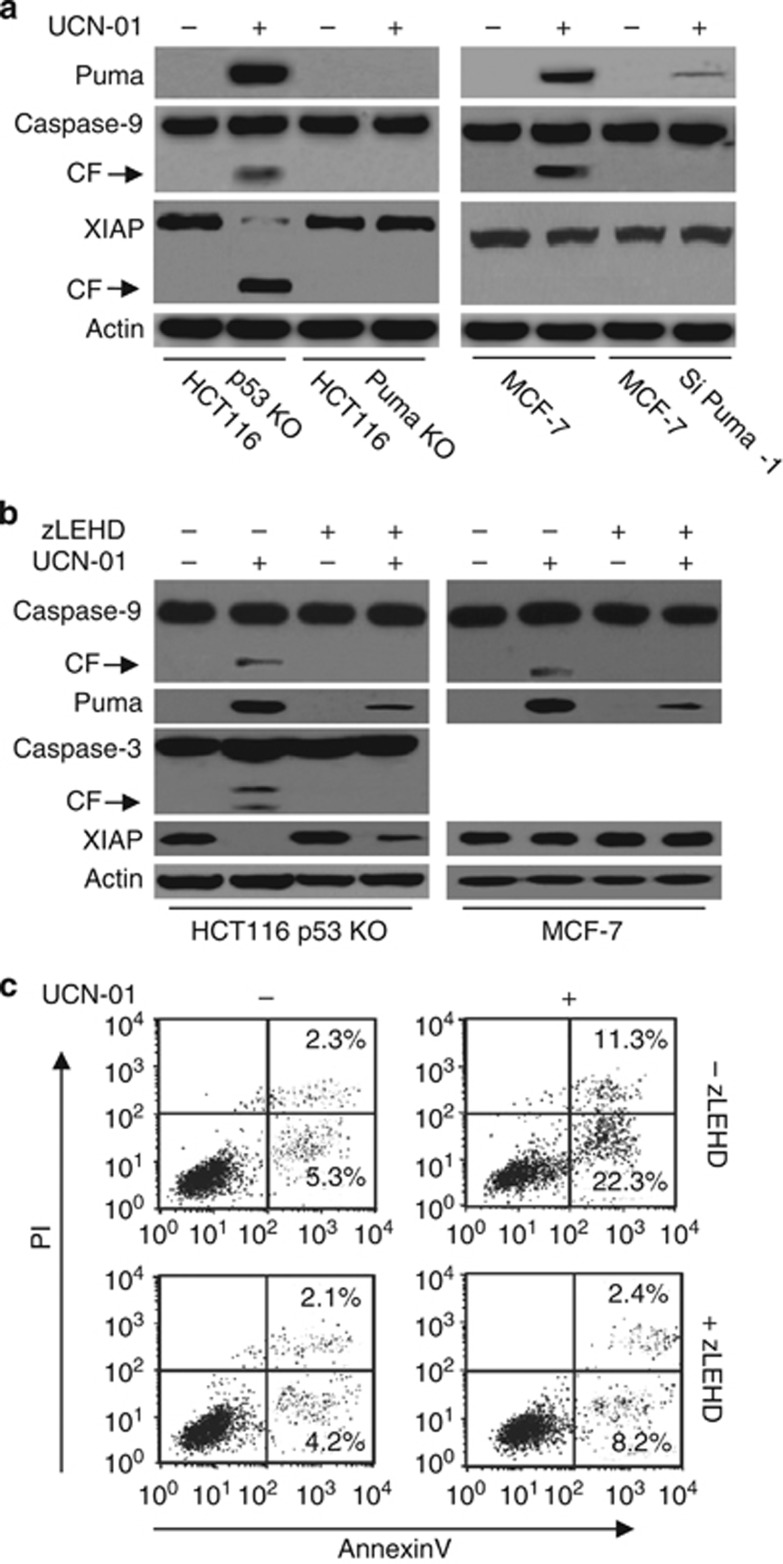
Caspase-9 regulates Puma-induced apoptosis. (**a**) HCT116 p53 KO, Puma KO, MCF-7 and MCF-7 Si Puma-1 cells were treated with 2 *μ*M UCN-01 for 48 h. Treated cells were collected and lysed for western blot analysis. *β*-Actin was used as a protein loading Ctrl. (**b**) Cells were pretreated with zLEHD (20 *μ*M) (a specific caspase-9 inhibitor) for 1 h, and then treated with 2 *μ*M UCN-01 for 48 h. Treated cells were collected and lysed for western blot analysis. *β*-Actin was used as a protein loading Ctrl. (**c**) As described in (**b**), cell apoptosis was detected with Annexin V/PI staining using flow cytometry

A recent study confirms that caspase-9 triggered caspase-3-independent apoptosis in UCN-01 treatment.^18^ Moreover, caspase-9 is critical for caspase-3 activation.^19^ We then examined whether caspase-9 contributes to Puma-induced apoptosis. We found that Puma indeed initiated caspase-9 activation (Figure 5a). Puma KO or KD by siRNA prevented caspase-9 cleavage (Figure 5a and Supplementary Figure 1B). Conversely, the caspase-9 inactivation by its inhibitor (zLEHD) decreased Puma expression after UCN-01 treatment (Figure 5b). Moreover, caspase-9 inhibition impeded caspase-3 activation. Flow cytometry assay revealed that caspase-9 inhibition decreased cell apoptosis (Figure 5c). We then used siRNA to interfere with caspase-9 expression. Caspase-9 siRNA (Si Casp-9-1) efficiently inhibited caspase-9 expression and caspase-3 cleavage in MEF and HCT116 p53 KO cells (Supplementary Figure 2A). Moreover, the inhibition of caspase-9 induction obviously decreased Puma expression and cell apoptosis (Supplementary Figures 2A and C). These results indicate that caspase-9 has a key role in Puma-induced apoptosis. Puma activates caspase-9 through the mitochondrial pathway. Caspase-9 then mediates the downstream apoptotic factor activation, such as caspase-3. Furthermore, when there is deficiency of caspase-3, caspase-9 positively regulates Puma expression through a certain feedback loop.

### Caspase-9 mediates Puma activation by cleaving Bcl-2 and Bcl-xL

**Figure 6:**
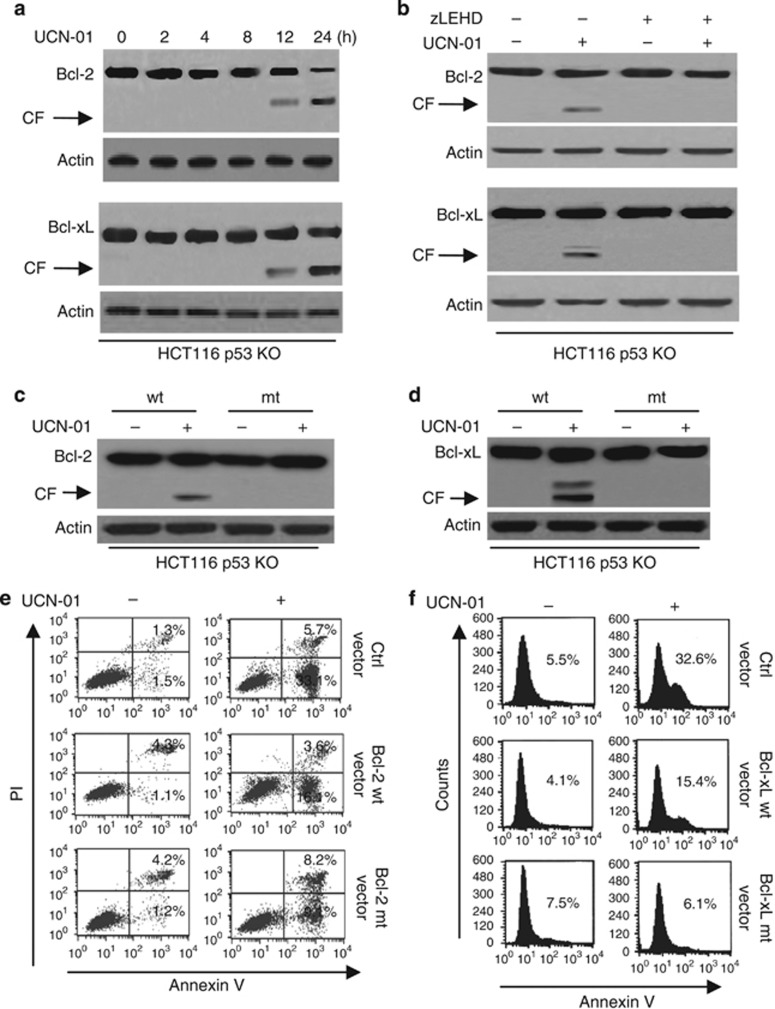
Caspase-9 degrades Bcl-2 and Bcl-xL to enhance Puma-induced apoptosis. (**a**) HCT116 p53 KO cells were treated with 2 *μ*M UCN-01 at different time points. Treated cells were collected and lysed for detection of Bcl-2 and Bcl-xL degradation. *β*-Actin was used as a protein loading Ctrl. (**b**) Cells were pretreated with zLEHD (20 *μ*M) (a specific caspase-9 inhibitor) for 1 h, and then treated with 2 *μ*M UCN-01 for 48 h. Treated cells were collected and lysed for detection of Bcl-2 and Bcl-xL degradation. *β*-Actin was used as a protein loading Ctrl. (**c**) HCT116 p53 KO cells were stably transfected with HA-Bcl-2 vector and then treated with 2 *μ*M UCN-01 for 48 h. Cells were lysed for detection of Bcl-2 degradation with anti-HA antibody. (**d**). As described in (**c**), cells were transfected with HA-Bcl-xL. Cells were lysed for detection of Bcl-xL degradation with anti-HA antibody. *β*-Actin was used as a protein loading Ctrl. (**e**) Cells were treated as described in (**c**), and then cell apoptosis was detected with Annexin V/PI staining using flow cytometry. (**f**) As described in (**d**), cells were stably transfected with HA-Bcl-xL vectors and cell apoptosis was detected with Annexin-positive staining

We then examined the feedback pathway that contributed to Puma activation. A previous study has pointed out that caspase-9 induces feedback apoptosis of the mitochondrion by the cleavage of antiapoptotic Bcl-2 and Bcl-xL.^20^ Our experiments proved that Bcl-2 and Bcl-xL was degraded during the time course of UCN-01 treatment (Figure 6a). Furthermore, caspase-9 inactivation restrained the cleavage of Bcl-2 and Bcl-xL (Figure 6b). Similarly, the inhibition of caspase-9 also prevented the degradation of Bcl-2 and Bcl-xL (Supplementary Figure 2A).

We next determined whether the cleavage of Bcl-2 and Bcl-xL is indeed involved in caspase-9-dependent apoptosis. We also generated the Bcl-2 or Bcl-xL mutant (D/A) as described previously.^20, 21, 22^ Wild-type (WT) or caspase-resistant mutant Bcl-2 or Bcl-xL was found to be stably transfected with HCT116 p53 KO cells. We examined the change of HA-Bcl-2 or HA-Bcl-xL transfectants. As expected, these mutants were resistant to degradation after UCN-01 treatment (Figures 6c and d). Overexpression of Bcl-2 or Bcl-xL potently inhibited caspase-9-induced apoptosis. WT Bcl-2 and Bcl-xL still induced cell death (Figures 6e and f).

### Caspase-3 regulates Puma activation and XIAP cleavage in apoptosis

We then examined the functional role of caspase-3 in Puma-induced apoptosis. We noted that caspase-9 activation resulted in the loss of XIAP in apoptosis (Figures 5a and b). However, when there is deficiency of caspase-3, the degradation of XIAP was stopped. These results suggest whether caspase-3 can regulate cell death through XIAP degradation.

**Figure 7:**
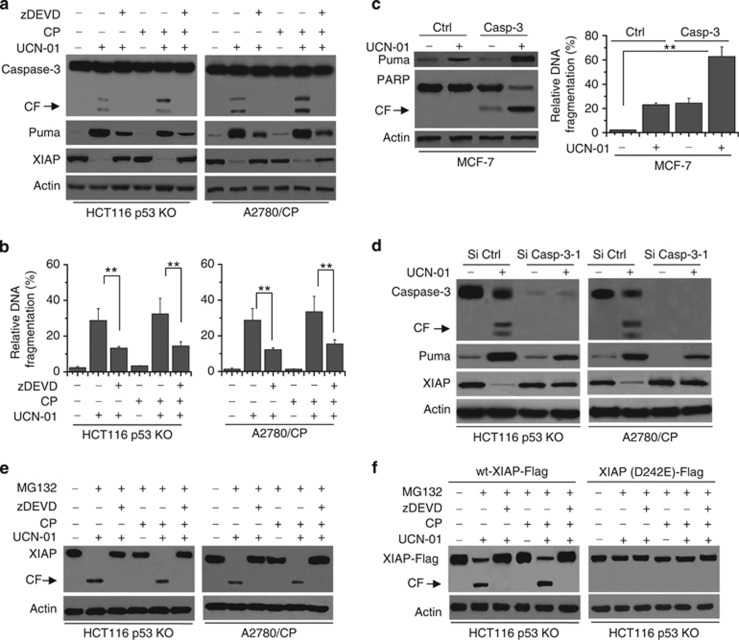
(**a**) Cells were pretreated with zDEVD (20 *μ*M) (a specific caspase-3 inhibitor) for 1 h, and then treated with 250 nM UCN-01, 100 nM UCN-01 and 15 *μ*M CP (for A2780/CP) or 2 *μ*M UCN-01, 500 nM UCN-01 and 15 *μ*M CP (for HCT116 p53 KO) for 48 h. Treated cells were collected and lysed for western blot analysis. *β*-Actin was used as a protein loading Ctrl. (**b**) As described in (**a**), cells were lysed to detect apoptosis by a Cell Death ELISA Kit as described in the Materials and Methods section. Graphs show results of quantitative analyses (*n*=3, mean±S.D., **P<0.01). (**c**) MCF-7 cells were transfected with Ctrl or full-caspase-3 vector for 48 h, and then treated with 2 *μ*M UCN-01 for 48 h. (Left) Treaded cells were collected and lysed for western blot analysis. (Right) Cells were lysed to detect apoptosis by a Cell Death ELISA Kit as described in the Materials and Methods section. Graphs show results of quantitative analyses (*n*=3, mean±S.D., **P<0.01). (**d**) HCT116 p53 KO and A2780/CP cells were transfected with si Ctrl or si Casp-3-1 for 48 h, and then treated with UCN-01 for 48 h. Treated cells were lysed for western blot analysis. (**e**) HCT116 p53 KO and A2780/CP cells were pretreated with zDEVD (20 *μ*M) and/or MG132 (the proteasome inhibitor) (20 *μ*M) for 1 h, and then treated with or without UCN-01, CP or their combination for 48 h for western blot analysis. (**f**) Cells were transfected with WT-XIAP-Flag or XIAP (D242E)-Flag vector for 48 h, and then pretreated with zDEVD and/or MG132 (20 *μ*M) for 1 h. Cells were treated with or without UCN-01, CP or their combination for 48 h for western blot analysis. All data are representative of three independent experiments

We first determined the effect of caspase-3 deficiency on Puma expression and cell death. We used HCT116 p53 KO and A2780/CP cells as cancer models. CP treatment was used as a control (Ctrl). We used the caspase-3 inhibitor (zDEVD) to specifically inhibit caspase-3 activity and found that caspase-3 inactivation decreased cell apoptosis after UCN-01 treatment alone, as well as with CP treatment. It is noteworthy that caspase-3 inactivation decreased Puma expression. Moreover, caspase-3 activation indeed resulted in the loss of XIAP in apoptosis after treatment (Figures 7a and b).

We transfected Ctrl or caspase-3 vector into MCF-7 cells and found that Puma expression increased following caspase-3 expression compared with Ctrl expression (Figure 7c). Moreover, caspase-3 induction resulted in PARP cleavage and apoptosis (Figure 7c). To further confirm the function of caspase-3 in Puma-induced apoptosis, we used siRNA to KD caspase-3 expression in HCT116 p53 KO and A2780/CP cells. Deficiency of caspase-3 decreased Puma expression and subsequently apoptosis. Meanwhile, caspase-3 inactivation led to the inhibition of XIAP degradation in apoptosis after treatment (Figure 7d and Supplementary Figure 3A).

To inquire whether XIAP depletion during apoptosis is dependent on proteasomal degradation, HCT116 p53 KO and A2780/CP cells were treated with the proteasome inhibitor MG132 before treatment. Proteasomal inhibition regained the truncated fragment of XIAP and no accumulation of the full-length XIAP could be detected, whereas zDEVD treatment could inhibit the cleaved fragment of XIAP and conserved the full-length XIAP (Figure 7e). These results were consistent with the reported study^23^ and suggest that caspase-3 cleaves XIAP into truncated fragment, which is subsequently committed to proteasomal degradation.

To confirm the effect of caspase-3 on XIAP, we transfected WT-XIAP-Flag or XIAP (D242E)-Flag into HCT116 p53 KO and A2780/CP cells. Ectopic expression of XIAP (D242E)-Flag variant in which the caspase cleavage motive was mutated prevented the depletion of XIAP compared with WT-XIAP-Flag (Figure 7f), and thus failed to show the cleaved product of XIAP following UCN-01 treatment upon proteasomal inhibition by MG132. Correspondingly, XIAP (D242E)-Flag appeared to be more potent in inhibiting apoptosis compared with WT-XIAP-Flag (data not shown). These results demonstrated that caspase-3 contributes to Puma-induced apoptosis, in which caspase-3 can cleave XIAP and positively regulate Puma activation to enhance apoptosis.

### Smac contributes to XIAP depletion, caspase processing and apoptosis enhancement

**Figure 8:**
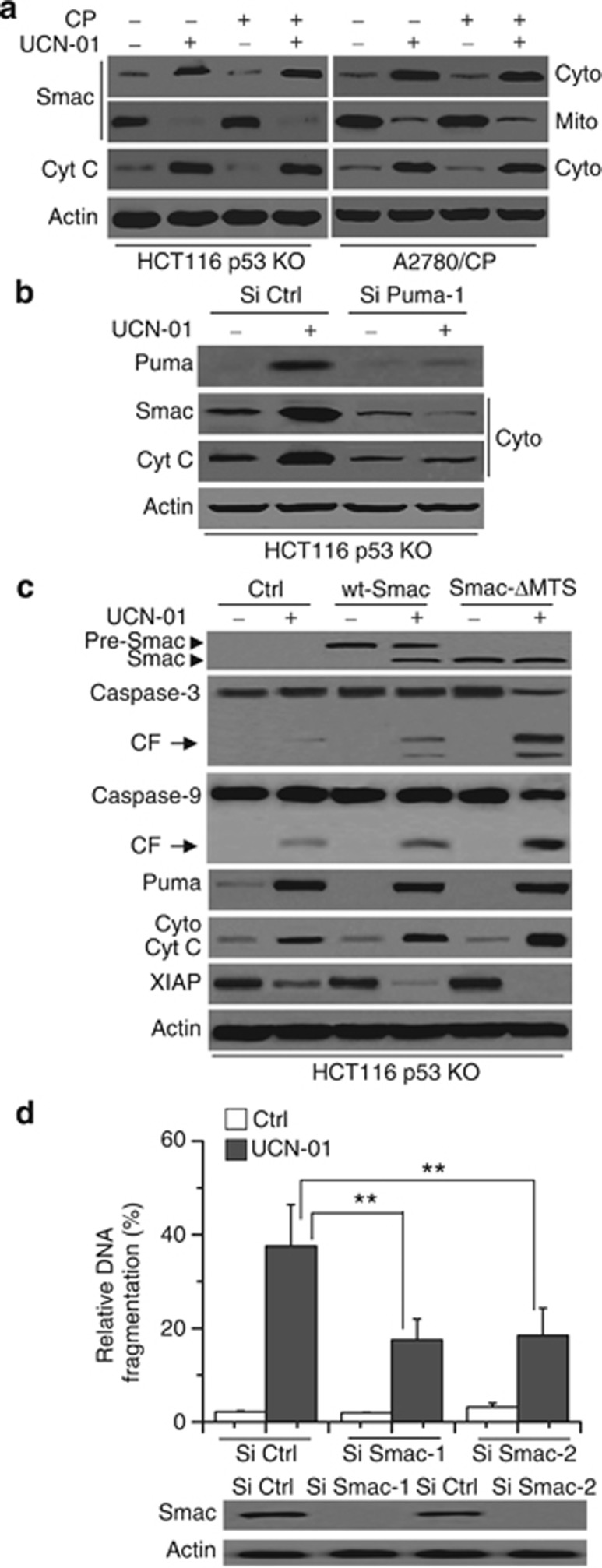
Smac contributes to XIAP depletion, caspase processing and apoptosis enhancement. (**a**) HCT116 p53 KO cells were treated with UCN-01, CP or their combination for 48 h as described in Figure 7 and subjected to subcellular fractionation. The cytosolic or mitochondrial fractions were immunoblotted. *β*-Actin was used as a protein loading Ctrl. (**b**) Cells were transfected with Si Ctrl or Si Puma-1 for 48 h. One part of treated cells was collected for western blot analysis, and another part of treated cells were subjected to subcellular fractionation. The cytosolic fractions were immunoblotted. (**c**) Cells were transfected with Ctrl, WT-Smac-Flag or Smac-ΔMTS-Flag for 48 h, and then cells were treated with 2 *μ*M UCN-01 for 48 h. One part of treated cells were collected for western blot analysis. Transfected Smac was detected by an anti-Flag antibody, and another part of treated cells were subjected to subcellular fractionation. The cytosolic fractions were immunoblotted. (**d**) Cells were transfected with Si Ctrl or Si Smac-1, 2 for 48 h, and then treated with 2 *μ*M UCN-01 for 48 h. Cells were lysed to detect apoptosis by a Cell Death ELISA Kit as described in the Materials and Methods section. Graphs show results of quantitative analyses (*n*=3, mean±S.D., **P<0.01)

We then detected whether second mitochondria-derived activator of caspase (Smac) is involved in UCN-01-induced apoptosis for it can connect with XIAP to release caspase-9 and -3.^23,24^ We used HCT116 p53 KO and A2780/CP cells as the example. CP treatment was used as a Ctrl. Other cells showed the same results (data not shown). Our data revealed that Smac was released from the mitochondria to cytosol after UCN-01 treatment (Figure 8a). Moreover, siPuma transfection inhibited Smac release and confirmed that Smac release is a downstream event of Puma activation (Figure 8b and Supplementary Figure 3B).

We next transfected Ctrl, WT-Smac-Flag or Smac-ΔMTS-Flag into HCT116 p53 KO cells. Smac-ΔMTS-Flag lacks the mitochondrial target sequence and locates in the cytosol.^23^ We found that both WT-Smac and truncated Smac expression enhanced caspase-9 and -3 cleavage to XIAP depletion (Figure 8c). Moreover, truncated Smac expression had more effect on caspase processing and XIAP degradation, as well as apoptosis, compared with WT-Smac. It was noteworthy that Smac expression had little effect on Cyt *c* release (Figure 8c), indicating that Smac does not mediate Cyt *c* activation in UCN-01-induced apoptosis. Further siRNA experiments revealed that Smac inhibition prevented UCN-01-induced apoptosis (Figure 8d) and demonstrated that Smac contributes to XIAP depletion, caspase processing and apoptosis enhancement.

### Puma and caspase-9 mediate the antitumor activity of UCN-01 in the xenograft models

To determine whether the caspase-9- and Puma-dependent apoptotic effect of UCN-01 contributes to its antitumor activity *in vivo*, we cloned the Puma siRNA (Si Puma-1) or Si Casp-9-1 into the pSilencer 2.1-U6 hygro plasmid to get Puma shRNA or Casp-9 shRNA, and later we stably transfected Puma shRNA or Casp-9 shRNA into HCT116 p53 KO or A2780/CP cells, respectively. Thus, we got different HCT116 p53 KO or A2780/CP cells (Supplementary Figures 4A and B).

**Figure 9:**
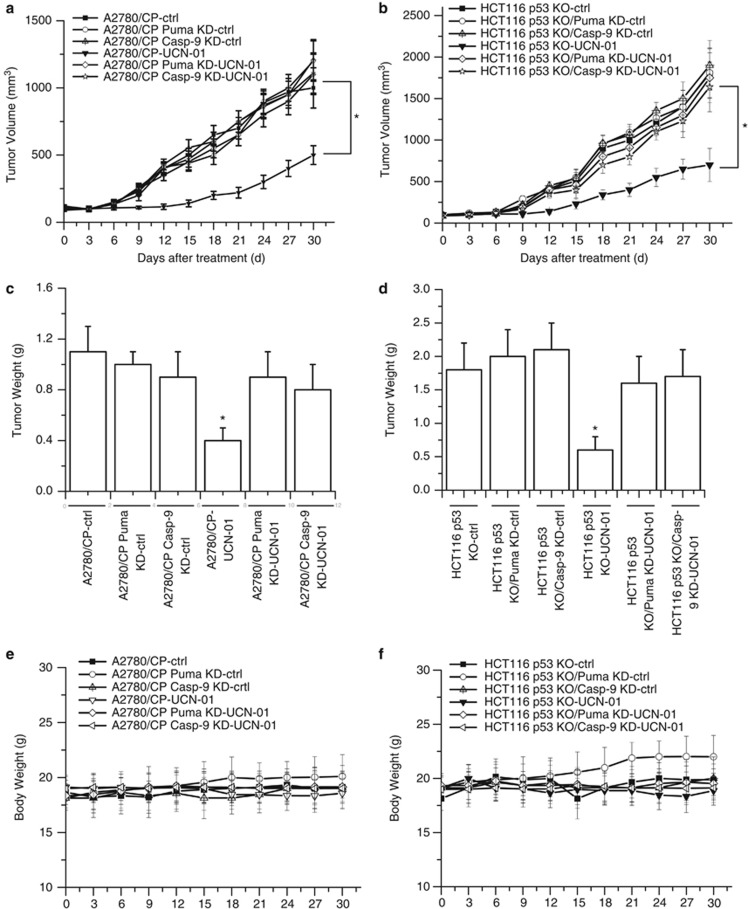
UCN-01 inhibits tumor growth *in vivo*. (**a**) The cell lines were subcutaneously injected into the right dorsal flank of 6- to 8-week-old female athymic nude BALB/c mice. Following tumor growth for 7 days, the tumor-bearing mice were randomly assigned into the following two groups (10 mice per treatment group): (**a**) Ctrl group and (**b**) UCN-01-treated group. Mice were injected intraperitoneally with UCN-01 as described in the Materials and Methods section. Tumor size was measured at the indicated days and volume was calculated (*P<0.05). (**b**) The cell lines were injected into nude mice and treated as described in (**a**). Tumor size was measured at the indicated days and volume was calculated (*P<0.05). (**c**) After the experiments are stopped, the tumor weight change of animals was measured. In A2780/CP tumor model, significant differences in tumor weight in mice treated with UCN-01 *versus* Ctrl are shown (*P<0.05). (**d**) As described in (**c**), the tumor weight of HCT116 p53 KO models was shown (*P<0.05). In the tumor weight of the other tumor model, no significant difference treated with UCN-01 *versus* Ctrl (*P*>0.05). (**e**) Body weights of A2780/CP mice models were plotted at 3-day intervals. There were no significant differences in weights among the different groups (*P*>0.05). Values were shown as mean±S.D. (**f**) As described in (**e**), body weights of HCT116 p53 KO mice models were shown. There were no significant differences in weights among the different groups (*P*>0.05). Values were shown as mean±S.D.

These cells were injected into the nude mice to establish xenograft tumors. Tumor-bearing mice were treated with UCN-01 as described previously,^25^ and tumor volumes were measured every 3 days for 30 days. We found that HCT116 p53 KO or A2780/CP tumors responded to UCN-01 treatment with slower growth and were generally half the size of the untreated tumors following treatment. In contrast, HCT116 p53 KO/Puma KD, HCT116 p53 KO/caspase-9 KD, A2780/CP/Puma KD or A2780/CP KO/caspase-9 KD tumors were indistinguishable from the untreated mice and did not respond to UCN-01 treatment (Figures 9a and b). The similar results were also found in the tumor weight (Figures 9c and d). To evaluate the possible adverse effects of UCN-01, the weight of the mice was monitored every 3 days throughout the whole experiment. The weight curve of UCN-01-treated group paralleled very closely with that of the Ctrl group (Figures 9e and f). No ruffled fur or toxic death was observed in the UCN-01-treated group.

**Figure 10:**
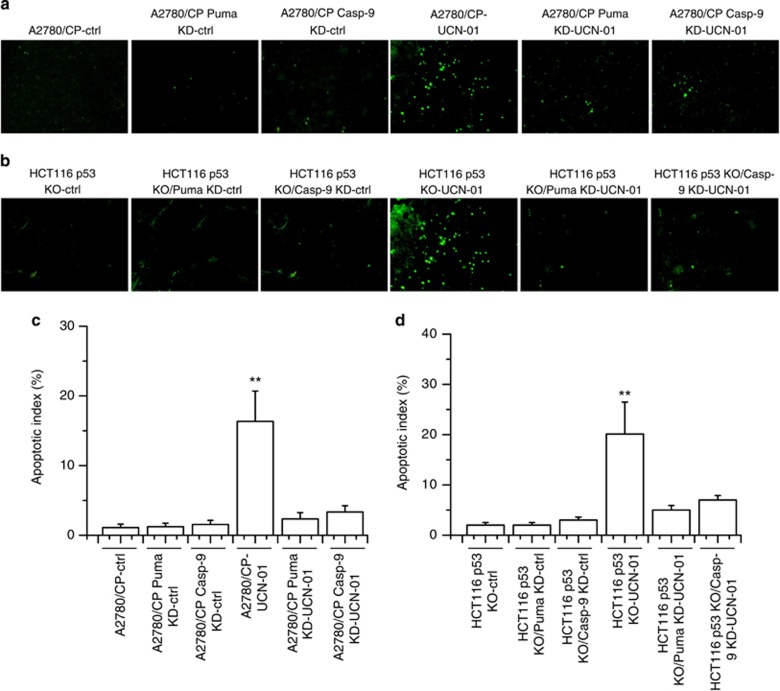
TUNEL assay in tumor models. Dissected tumors were weighed and fixed in 4% paraformaldehyde in PBS, embedded in paraffin and cut into 3–5 *μ*m sections. The sections were used for TUNEL experiments. Tumor tissue preparation and procedure for TUNEL staining is described in the Materials and Methods section. (**a** and **b**) The detection of apoptotic cells using TUNEL analysis in different HCT116 p53 KO or A2780/CP tumor models. Representative sections were shown. (**c** and **d**) The apoptotic index in different tumor models was shown. Graphs show the apoptotic results of quantitative analyses. In different HCT116 p53 KO or A2780/CP tumor model, significant differences in apoptotic index in tumor treated with UCN-01 *versus* Ctrl are shown (**P<0.01). In the other tumor model, no significant difference in apoptotic index treated with UCN-01 *versus* Ctrl (*P*>0.05)

Tumor cell apoptosis was assessed by terminal deoxynucleotidyltransferase-mediated dUTP nick-end labeling (TUNEL) assay. As shown in Figures 10a and b, a significantly greater percentage of TUNEL-positive nuclei could be observed in HCT116 p53 KO or A2780/CP tumors treated with UCN-01 when compared with the tumors from the Ctrl group (Figures 10a and b). However, HCT116 p53 KO/Puma KD, HCT116 p53 KO/caspase-9 KD, A2780/CP/Puma KD or A2780/CP KO/caspase-9 KD tumors revealed almost the same percentage of TUNEL-positive nuclei with that of the Ctrl group (Figures 10c and d). These data clearly show the necessity of Puma and caspase-9 for the *in vivo* antitumor and apoptotic effects of UCN-01.

## Discussion

The previous study had revealed that UCN-01 could induce Puma-related apoptosis in a p53-independent manner.^25^ In this study, we proved these results and further provided the evidence that UCN-01 could induce Puma expression in other cancer cells with dysfunctional p53 such as A2780/CP^7^ and K526.^26^ Moreover, our data demonstrated that UCN-01 triggered Puma induction in CP-resistant cancer cells (A2780/CP and K526/CP) and confirmed that UCN-01 could function as a chemosensitizer. UCN-01, which is the broad-range kinase inhibitor, has additive or even synergistic effects on apoptosis induced by a variety of commonly used chemotherapeutic drugs^25^ and is being evaluated in several clinical trials (http://clinicaltrials.gov). The previous study revealed that MEK inhibitors potentiated UCN-01-mediated apoptosis through a Bim-dependent manner.^15^ Both our and Dudgeon *et al.*'s report^25^ demonstrated that UCN-01 could trigger Puma-related apoptosis. Therefore, these results suggest that UCN-01 can induce different apoptosis pathway in different cell lines. However, the one definite thing is that UCN-01 can modulate BH3-only proteins to potentiate apoptosis in cancer cells.

Our recent study provides the evidence that JNK and Akt corporately mediates Puma induction in apoptosis.^7^ In this study, we also confirmed that Akt-FoxO3a regulates Puma expression. However, although JNK was activated in UCN-01-induced apoptosis, we found that JNK was not involved in regulating Puma expression. JNK inhibitor SP600125 inhibited JNK activation but had little effect on Puma induction and apoptosis (data not shown). These results indicate that different agents may allow for simultaneous Puma induction via multiple pathways; therefore, lowering the threshold proapoptotic activity is required for apoptosis induction.^8^ Of course, we still need to perform further experiments to study the reasons why JNK is not involved in apoptosis induced by UCN-01 treatment in our study.

As far as we know, Puma initiates apoptosis mainly through the mitochondrial apoptotic pathway, such as Cyt *c* release and subsequent caspase activation.^6^ In this study, our data indeed revealed that Puma triggered this apoptotic pathway. However, interestingly, Puma still induced apoptosis in MCF-7 cells, which is caspase-3 deficient.^17^ The result confirms at least two things. First, caspase-3 participates in the process of apoptosis, but cannot play a decisive role. Second, the other pathway is involved in Puma-induced cell death.

The recent study has proved that caspase-9 contributes to UCN-01-treated apoptosis. Caspase-9 induced an Apaf-1-independent apoptosis in MCF-7 cells after UCN-01 treatment.^18^ We speculate that caspase-9 has a key role in Puma-induced apoptosis after UCN-01 treatment. Our data demonstrated that Puma initiated caspase-9 activation. Moreover, caspase-9 not only participates in Puma-induced apoptosis but also regulates Puma expression through cleaving Bcl-2 and Bcl-xL. Caspase-9 inactivation efficiently inhibited Puma expression, caspase-3 cleavage and cell death. Therefore, our results may elaborate the molecular mechanism of Apaf-1-independent caspase-9 activation in apoptosis. After UCN-01 treatment, activated Puma mediates Cyt *c* release and subsequently caspase-9 activation. Caspase-9 as a central molecule transmits the apoptotic signal in cell death. On the one hand, caspase-9 enhances Puma activation by feedback amplification independent of caspase-3. Puma activation could induce the release of AIF and Endo G,^27,28^ which induce caspase-3-independent cell death.^28^ On the other hand, caspase-9 also triggered caspase-3 signaling pathway in the presence of caspase-3.

Our data also revealed that caspase-3 could mediate Puma-induced apoptosis through a feedback loop. The previous study demonstrated that caspase-3 regulate Cyt *c* release and Bak activation by a feedback amplification loop.^6,29^ It is possible that caspase-3 also unleashes further mitochondrial changes that are necessary for full execution of apoptotic events to enhance Puma activation. Indeed, Puma induction triggered Smac release from the mitochondria and Smac induced distraction of XIAP from caspase-3 to result in the initial activation of caspase-3. Activated caspase-3 then cleaved XIAP for proteasomal degradation, thereby initiating a positive amplification loop causing enhanced apoptosis.

In conclusion, we explore, for the first time, the detailed molecular mechanisms of Puma-induced apoptosis after UCN-01 treatment and demonstrate that caspase-9 is indispensable for UCN-01-induced apoptosis. Our findings can explain why UCN-01 can induce cell apoptosis in chemoresistant cancer cells. Puma initiates downstream caspase processing. Caspase-9 and-3 could reinforce Puma activation and apoptosis through two positive amplification loops. These positive regulatory feedback loops sensitizes cancer cells to treatment with UCN-01. It seems to have broader implications, even in a clinical perspective.

## Materials and Methods

### Materials

UCN-01, PI, the proteasome inhibitor MG132 and CP were obtained from Sigma (St. Louis, MO, USA). zDEVD-fmk and zLEHD-fmk were from BD Biosciences (San Jose, CA, USA). Flag (clone M1, no. F3040) and actin (clone AC-74, no. A5316) antibodies were purchased from Sigma. Puma (no. 4976), PARP (clone 46D11, no. 9532), XIAP (no. 2042), Smac (no. 2954), p-Akt (Ser 473) (clone 587F11, no. 4051), Akt (no. 9272), caspase-3 (clone 8G10, no. 9665), HA (clone 6E2, no. 2367) and caspase-9 (clone C9, no. 9508) antibodies were purchased from Cell Signaling (Beverly, MA, USA). Cyt *c* (sc-13156), Bcl-2 (sc-7382) and Bcl-xL (sc-8392) antibodies were from Santa Cruz (Santa Cruz, CA, USA). Lamin B1 (ab16048) antibody was from Abcam (Cambridge, UK). FoxO3a (07-702) were from Upstate Biotechnology (Lake Placid, NY, USA).

### Gene silencing with small interfering RNAs and plasmids

siRNA oligonucleotides were purchased from Dharmacon (Lafayette, CO, USA) with sequences targeting Smac-1 (5′-AACCCUGUGUGCGGUUCCUAU-3′), Smac-2 (5′-CCACAUAUGCGUUGAUUGAAGCUAU-3′); Puma-1 (5′-UCUCAUCAUGGGACUCCUG-3′), Puma-2 (5′-CAGUGGGCCCGGGAGAUCG-3′); FoxO3a-1 (5′-ACUCCGGGUCCAGCUCCAC-3′), FoxO3a-2 (5′-GAGCUCUUGGUGGAUCAUC-3′); caspase-3-1 (5′-UGAUCUUACACGUGAAGAATT-3′), caspase-3-2 (5′-UGAGGUAGCUUCAUAGUGGTT-3′); and caspase-9-1 (5′-CCAGGCAGCUGAUCAUAGA-3′), caspase-9-2 (5′-CGACCUGACUGCCAAGAAA-3′). For Puma, caspase-9 shRNA construction, the Si Puma-1 or Si Casp-9-1 was cloned into the pSilencer 2.1-U6 hygro plasmid. The constitutively active Akt1 construct HA-PKB-T308D/S473D was obtained as described previously.^30^ Smac and XIAP constructs were generated by RT–PCR from total RNA isolated from cells and cloning of the RT–PCR products into the pFlag-CTC vector (Sigma). HA-Bcl-2 and HA-Bcl-xL were gifts from David CS Huang (Walter And Eliza Hall Institute For Medical Research, Melbourne, VIC, Australia). The non-cleavable D242E mutant form of XIAP, Smac-ΔMTS HA-Bcl-2 (D/A) and HA-Bcl-xL (D/A) were generated by site-directed mutagenesis using *Pfu*-ultra polymerase (Stratagen, La Jolla, CA, USA) followed by *Dpn*I digestion (Fermentas Inc., Glen Burnie, MD, USA) according to the manufacturer's instructions. Ctrl and Full-caspase-3 vector is obtained from Andreas Marti (University of Bern, Bern, Switzerland).

### Cell culture and transfection

K562/S (CP-sensitive) (p53 mutant), MCF-7 (caspase-3 mutant), MEFs, MDA-MB-231, HCT116, HT29 and DLD1 (both p53 mutant) cells were obtained from the American Type Culture Collection (ATCC, Manassas, VA, USA). CP-sensitive (A2780/S) and -resistant (A2780/CP) human ovarian cancer cell lines were obtained as described previously.^7^ Cells were cultured with DMEM media (Sigma) supplemented with 10% fetal bovine serum (Hyclone, Logan, UT, USA) and 1% penicillin–streptomycin at 37 °C under 5% CO_2_. CP-resistant cells (A2780/CP and K526/CP) were obtained by iterative treatments with increasing concentrations of CP as described previously.^31^ HCT116 p53 KO and Puma KO cells are the gifts from Dean G Tang (The University of Texas MD Anderson Cancer Center, Science Park-Research Division, Smithville, TX, USA).

For transfection, cells were seeded on 6-well plates and then transfected with the appropriate plasmid DNA or siRNA using the manufacturer's protocols. Typically, cells were seeded on coverslips in the 6-well plates, and then 1 *μ*g of plasmid DNA or 100 nM siRNA and 4 *μ*l of DMRIE-C reagent (Invitrogen, Carlsbad, CA, USA) were used per coverslip. The cells were incubated for 4 h in the transfection mixture, which was then replaced with fresh culture medium.

### Cell viability and apoptosis assays

Four methods were used to assess AT101-induced apoptotic cell death: detection of DNA fragmentation with the Cell Death Detection ELISA Kit (Roche Diagnostics, Basel, Switzerland), western blot analysis of caspase activation, PARP cleavage and measurement of apoptotic cells by flow cytometry (PI staining for sub-G1 or AnnexinV/PI). The Cell Death Detection ELISA Kit quantified the apoptotic cells by detecting the histone-associated DNA fragments (mono- and oligonucleosomes) generated by the apoptotic cells as described previously.^30^

### Cell fractionation and immunoblotting

Mitochondrial and cytoplasmic cell fractions were obtained by differential centrifugation as described previously.^30^ Nuclear and cytoplasmic lysates were prepared according to a previously published protocol.^7^ Immunoblotting was carried out as described previously.^31^ Western blot was carried out with antibody dilutions as follows: actin at 1 : 20 000; Puma, XIAP, Flag, p-Akt (Ser 473), Akt, caspase-3, HA, caspase-9, Bcl-2 and Bcl-xL at 1 : 2000; and PARP, FoxO3a, Cyt *c*, Smac, Lamin B1 at 1 : 1000.

### ChIP assay

The ChIP assay was performed using the Chromatin Immunoprecipitation Assay Kit (Upstate Biotechnology, Lake Placid, NY, USA) according to the manufacturer's protocol. ChIP was performed with 3 *μ*g of FoxO3a antibody (Sigma) incubated with protein G-coated magnetic beads overnight with rotation at 4 °C. The DNA fragments that co-immunoprecipitated with the target proteins FoxO3a were subjected to quantitative real-time PCR (QRT-PCR) analysis using various primer sets. The Ct value of each sample was normalized to the Ct value obtained from the PCR reaction using the corresponding input genomic DNA as a template. Primer sequences for Puma and FoxO3a binding were designed as described previously.^7^ Primer sequences used were as follows: Puma FoxO3a, 5′-GCCGCCACTGCAGTTAGAG-3′ and 5′-AACAGCCGGTTATTGGCC-3′.

### Immunostaining

The experiments were performed according to our previous report.^30^ MEF cells were seeded in 24-well plates with Lab-Tek Chamber Slides with a Cover (Nalge Nunc International, Naperville, IL, USA) in 500 *μ*l medium and incubated overnight. Cells were then treated with UCN-01 (6 *μ*M) for 48 h. The medium was removed and cells were fixed in 4% formaldehyde containing 0.1% glutaraldehyde for 15 min at room temperature (RT). After rinsing with cold PBS (pH 7.4), cells were permeabilized with 0.5% Triton X-100 for 10 min at RT. After blocking with 5% goat serum, Cyt *c* (7H8.2C12; BD Pharmingen, San Diego, CA, USA) (1 : 100 dilution) was added, and the fixed cells were incubated with antibodies at 37 °C for 1 h, followed by incubation with anti-mouse IgG–Cy5 (Millipore, Boston, MA, USA; 1 : 128 dilution) for 1 h. After the removal of antibodies, cells were rinsed with PBS and mounted with 90% glycerol. Fluorescence was immediately observed using an Olympus DP72 microscope (Olympus Corporation, Tokyo, Japan).

### *In vivo* tumor experiments

*In vivo* experiments were performed according to our previous report with some modifications.^8,32^ To study the antitumor activities of UCN-01 *in vivo*, HCT116 p53 KO and A2780/CP models were established. We cloned the Si Puma-1 or Si Casp-9-1 into the pSilencer 2.1-U6 hygro plasmid to get Puma shRNA or Casp-9 shRNA, and later we transfected Puma shRNA or Casp-9 shRNA into HCT116 p53 KO or A2780/CP, respectively. We screened the cell lines to obtain stably transfected cell lines. Thus, we obtained HCT116 p53 KO/Puma KD, HCT116 p53 KO/caspase-9 KD, A2780/CP/Puma KD or A2780/CP /caspase-9 KD. In brief, 4 × 10^6^ different HCT116 p53 KO cell lines or 2.5 × 10^6^ different A2780/CP cell lines were subcutaneously injected into the right dorsal flank of 6- to 8-week-old female athymic nude BALB/c mice. Following tumor growth for 7 days, the tumor-bearing mice were randomly assigned into the following two groups (10 mice per treatment group): (a) Ctrl group; (b) UCN-01-treated group. Mice were injected intraperitoneally for 5 consecutive days with 9 mg/kg UCN-01 diluted in 20 mmol/l sodium citrate buffer (pH 6).^25^ Tumor volumes were evaluated according to the following formula: tumor volume (mm^3^)=0.52 × length × width^2^. The weight of the mice was measured at 3-day intervals. At the end of the experiment, the mice were killed. Tumor net weight of each mouse was measured. The tumor tissues were collected for subsequent TUNEL experiments (see below). All studies involving mice were approved by the Institutional Animal Care and Treatment Committee of Sichuan University (Chengdu, China).

### TUNEL assay

The presence of apoptotic cells within the tumor sections was evaluated by the TUNEL technique using the DeadEnd Fluorometric TUNEL System (Promega, Madison, WI, USA) following the manufacturer's protocol. Percent apoptosis was determined by counting the number of apoptotic cells and dividing by the total number of cells in the field (5 high power fields/slide).

### Statistical analysis

Statistical analysis of the differences between the groups was performed using the Student's *t*-test with *P*<0.05 considered statistically significant.

## Abbreviations

Cyt c: cytochrome c
Smac: second mitochondria-derived activator of caspase
p-Akt: the phosphorylation level of Akt
p-FoxO3a: the phosphorylation level of FoxO3a
