# Supplementary material for: Caspase-9 mediates Puma activation in UCN-01-induced apoptosis
Source: Cell Death Dis. 2014 Oct 30;5(10):e1495–. doi: 10.1038/cddis.2014.461 (PMC4649536; doi:10.1038/cddis.2014.461)
Supplement: Supplementary Figure legend [file cddis2014461x1.doc]

**Supplementary Figure legends**

Supplementary Fig.1. **A.** HCT116 p53 KO cells were transfected with Si Ctrl or Si FoxO3a-2 for 48 h, and then treated with 2 µM UCN-01 for 48 h. Treated cells were collected and lysed for Western blot analysis. **B.** MCF-7 cells were transfected with Si Ctrl or Si Puma-2 for 48 h, and then treated with 2 µM UCN-01 for 48 h. Treated cells were collected and lysed for Western blot analysis. **C.** As described in **B**, MCF-7 cells were treated and cell apoptosis were examined. Graphs showing results of quantitative analyses (*n*=3, mean ± S.D. **, *P*<0.01).

Supplementary Fig.2. Caspase-9 siRNA obviously inhibits Puma-induced apoptosis. **A.** MEF or HCT116 p53 KO cells were transfected with Si Ctrl, Si-Casp-9-1, 2 for 48h, and then treated with UCN-01 for 48 h. Cells were collected and lysed for Western blot analysis. **B.** As described in **A**, MEF cells were treated and cell apoptosis were examined. Graphs showing results of quantitative analyses (*n*=3, mean ± S.D. **, *P*<0.01). **C.** As described in **A**, HCT116 p53 KO cells were treated and cell apoptosis were examined. Graphs showing results of quantitative analyses (*n*=3, mean ± S.D. **, *P*<0.01).

Supplementary Fig.3. **A.** HCT116 p53 KO cells were transfected with Si Ctrl or Si Casp-3-2 for 48 h, and then treated with 2 µM UCN-01 for 48 h. Treated cells were collected and lysed for Western blot analysis. **B.** HCT116 p53 KO cells were transfected with Si Ctrl or Si Puma- 2 for 48 h, and then treated as described in **A**. cells were collected and lysed for Western blot analysis. β-Actin was used as a protein loading control.

Supplementary Fig.4. **A.** Cells were transfected with Ctrl shRNA or Puma shRNA and screened to get stable transfected cell lines. Cells were collected and lysed for detecting Puma expression. **B.** Cells were transfected with Ctrl shRNA or Casp-9 shRNA and screened to get stable transfected cell lines. Cells were collected and lysed for detecting caspase-9 expression. β-Actin was used as a protein loading control.
