# Supplementary figures and images for: Caspase-9 mediates Puma activation in UCN-01-induced apoptosis
Source: Cell Death Dis. 2014 Oct 30;5(10):e1495–. doi: 10.1038/cddis.2014.461 (PMC4649536; doi:10.1038/cddis.2014.461)

Supplementary Fig. 1

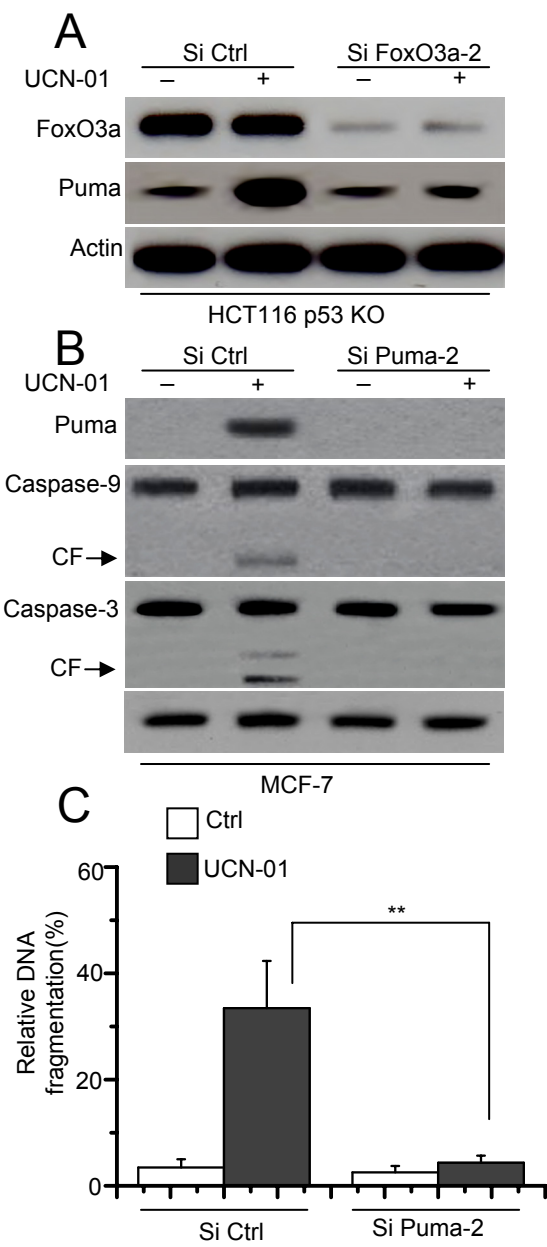

Supplementary Fig. 2

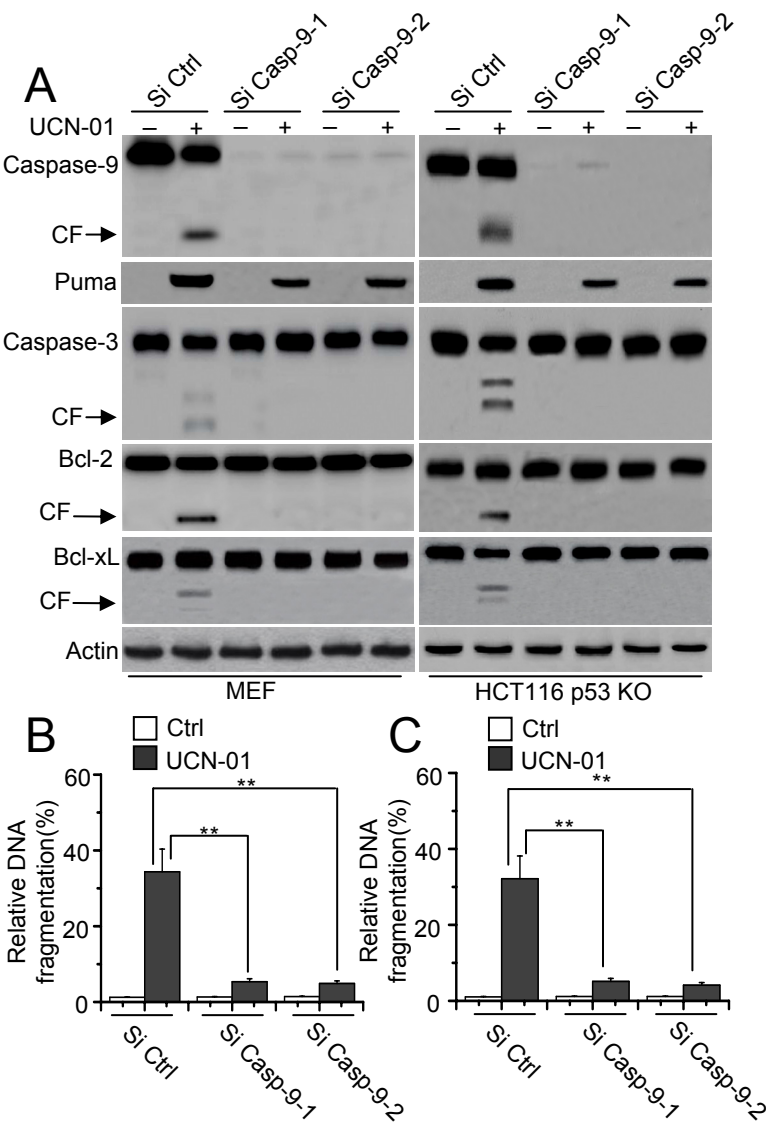

Supplementary Fig. 3

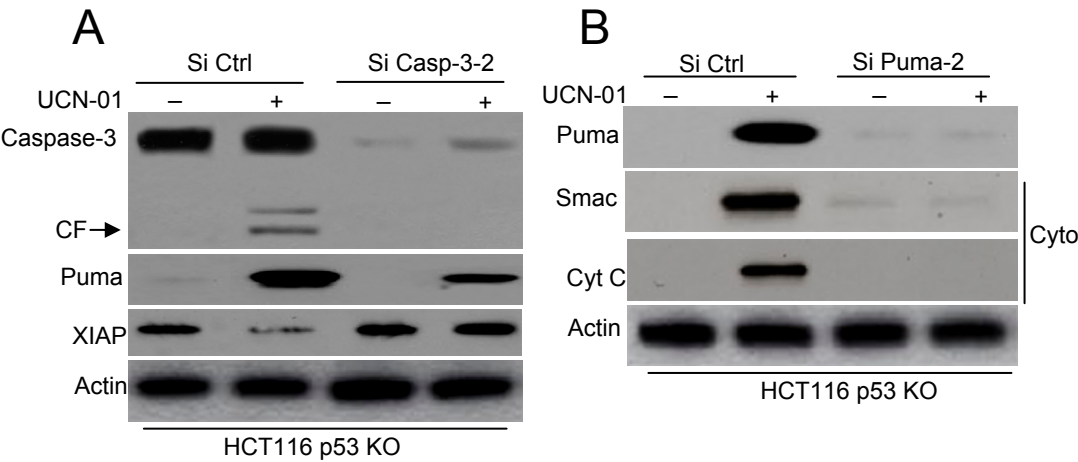

Supplementary Fig. 4

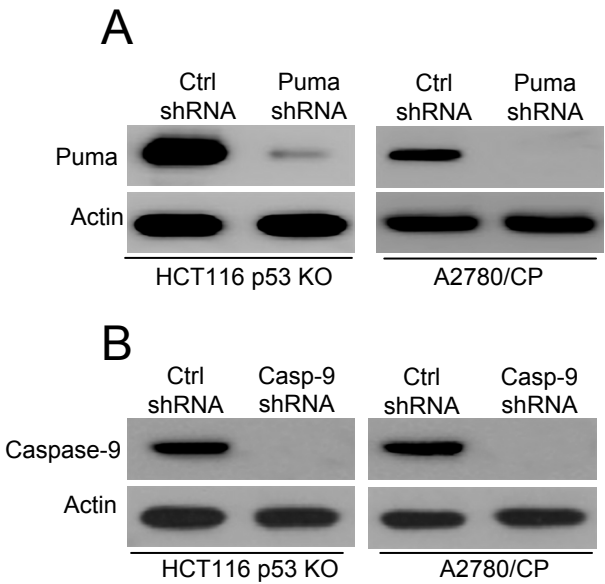

Supplement: Supplementary Figures [file cddis2014461x2.pdf]
